# The Impact of Implementing a Test, Treat and Retain HIV Prevention Strategy in Atlanta among Black Men Who Have Sex with Men with a History of Incarceration: A Mathematical Model

**DOI:** 10.1371/journal.pone.0123482

**Published:** 2015-04-23

**Authors:** Viviane D. Lima, Isabell Graf, Curt G. Beckwith, Sandra Springer, Frederick L. Altice, Daniel Coombs, Brian Kim, Lauren Messina, Julio S. G. Montaner, Anne Spaulding

**Affiliations:** 1 British Columbia Centre for Excellence in HIV/AIDS, Vancouver, British Columbia, Canada; 2 Department of Medicine, Division of AIDS, Faculty of Medicine, University of British Columbia, Vancouver, British Columbia, Canada; 3 Department of Mathematics, Simon Fraser University, Burnaby, Canada; 4 Alpert Medical School of Brown University, The Miriam Hospital, Providence, Rhode Island, United States of America; 5 Yale University School of Medicine, Section of Infectious Diseases, AIDS Program, New Haven, Connecticut, United States of America; 6 Yale University School of Public Health, Division of Epidemiology of Microbial Diseases, New Haven, Connecticut, United States of America; 7 Department of Mathematics, University of British Columbia, Vancouver, British Columbia, Canada; 8 Rollins School of Public Health Department of Epidemiology, Emory University School of Medicine, Atlanta, United States of America; University of New South Wales, AUSTRALIA

## Abstract

**Background:**

Annually, 10 million adults transition through prisons or jails in the United States (US) and the prevalence of HIV among entrants is three times higher than that for the country as a whole. We assessed the potential impact of increasing HIV Testing/Treatment/Retention (HIV-TTR) in the community and within the criminal justice system (CJS) facilities, coupled with sexual risk behavior change, focusing on black men-who-have-sex-with-men, 15–54 years, in Atlanta, USA.

**Methods:**

We modeled the effect of a HIV-TTR strategy on the estimated cumulative number of new (acquired) infections and mortality, and on the HIV prevalence at the end of ten years. We additionally assessed the effect of increasing condom use in all settings.

**Results:**

In the Status Quo scenario, at the end of 10 years, the cumulative number of new infections in the community, jail and prison was, respectively, 9246, 77 and 154 cases; HIV prevalence was 10815, 69 and 152 cases, respectively; and the cumulative number of deaths was 2585, 18 and 34 cases, respectively. By increasing HIV-TTR coverage, the cumulative number of new infections could decrease by 15% in the community, 19% in jail, and 8% in prison; HIV prevalence could decrease by 8%, 9% and 7%, respectively; mortality could decrease by 20%, 39% and 18%, respectively. Based on the model results, we have shown that limited use and access to condoms have contributed to the HIV incidence and prevalence in all settings.

**Conclusions:**

Aggressive implementation of a CJS-focused HIV-TTR strategy has the potential to interrupt HIV transmission and reduce mortality, with benefit to the community at large. To maximize the impact of these interventions, retention in treatment, including during the period after jail and prison release, and increased condom use was vital for decreasing the burden of the HIV epidemic in all settings.

## Introduction

In the United States (US), at the end of 2011, it was estimated that 1.1 million people were living with HIV/AIDS (PLWHA), and approximately 50,000 individuals became newly infected yearly [1–4]. The HIV epidemic is very heterogeneous depending on race, age, neighborhood, gender and other social and economic factors [1, 4]. Among PLWHA, 78% were males, 44% were African-Americans and 62% were between 35 and 54 years [1–3]. Men-who-have-sex-with-men (MSM) (62%) contributed the most to PLWHA, followed by high-risk heterosexual contact (28%) and injection drug use (8%) [1–3]. By year-end 2010, Georgia ranked sixth among states in terms of HIV/AIDS prevalence rate (429 per 100,000 population) [3].

In parallel, the US is also facing a serious HIV epidemic within the criminal justice system (CJS) [4, 5]. At year-end 2010, there were 20,093 PLWHA among the 2.3 million adults incarcerated (prevalence 0.87%), including 1,578 federal and 18,515 state prisoners. HIV prevalence among those within the US-CJS is three times greater than those within the community, with Georgia ranking seventh in the US [5]. Persons of color, especially of black race (mostly African-American), are disproportionately affected by HIV and incarceration compared to their Caucasian counterparts [6, 7]. Though HIV can be transmitted within US-CJS settings through forced or consensual sex (facilitated by limited condom availability) [8–10], injection drug use (facilitated by the absence of sterile injecting equipment and near absence of evidence-based drug treatment)[10], and tattooing [10, 11], most HIV transmission occurs outside the CJS. Thus, understanding the dynamics of HIV among persons cycling in and out of correctional facilities is important for developing community-wide HIV prevention strategies.

Over the last decade, expanded HIV testing, antiretroviral (ART) treatment and retention in long-term treatment (HIV-TTR) has been proposed as a powerful strategy to reduce HIV-related morbidity and mortality and secondarily decrease HIV transmission [12–15]. Studies have shown that by decreasing plasma HIV-1 RNA levels to undetectable levels (typically <50 copies/mL) in blood, semen and vaginal secretions on a long-term basis can dramatically reduce HIV transmission [12, 16]. Since then, several groups, with exception of MSM communities in Australia, Amsterdam and in the UK, have shown that for any given increase in ART coverage a subsequent decrease in HIV transmission can be expected [12, 17].

In the present study, we sought to develop a mathematical transmission model to predict the community-wide impact of intensifying prevention interventions both within in the community and CJS facilities, including expansion of HIV-TTR and sexual risk behavior changes. The choice of focusing this model on the CJS in Fulton County, Atlanta, Georgia, was driven by two facts: (1) the management of inmates across CJS facilities in different states in the US vary dramatically, and by focusing on only one state we minimized biases that could be introduced in our analyses; and (2) we have access to a selection of rich data sources. Fulton County is an urban county, which comprises 90% of the city of Atlanta and the most populated county in Georgia, with very high incarceration rates and HIV incidence and prevalence. In 2012, there were 26,344 male inmates in Fulton County Jail and 51,868 male inmates confined in all Georgia state prisons. Demographically, Black males are at the highest risk of CJS involvement and HIV infection in Fulton County. In addition, MSM transmission contributes most to PLWHA in Fulton County for males (91% of new infections); and heterosexual transmission accounts for 87% of new infections in females) [1–3, 18, 19]. Injection drug use is relatively rare in Fulton County [19]. To assess the impact of a CJS-based HIV-TTR intervention, this model was based upon 2012 Fulton County data including persons aged 15–54 years, of black race, and whose risk for acquiring HIV is MSM through unprotected anal intercourse.

## Materials and Methods

### Data

Data for population estimates, population growth, HIV/AIDS and non-HIV/AIDS mortality were obtained from the US Census Bureau, the US Centers for Disease Control (CDC), and the Georgia Department of Public Health [1–3, 6, 19–30]. HIV testing data in the CJS were obtained from the IIDDEAL study, which was a US CDC demonstration project in Fulton County Jail [18]. Longitudinal data for the jail setting for ART uptake and retention in treatment were obtained from the EnhanceLink project, which was a Special Projects of National Significance funded by the Health Resources and Service Administration HIV/AIDS Program [31–36]. Longitudinal data for the prison setting for HIV testing, ART uptake and retention in treatment were obtained from the Georgia Department of Corrections and published literature [37–39]. The size of the incarcerated population and HIV/AIDS and non-HIV/AIDS mortality data in the CJS were obtained from the Bureau of Justice Statistics and the National Corrections Reporting Program [5, 6, 29, 30, 37, 40–43]. We also incorporated published data on HIV testing, ART uptake and retention in treatment at the community-level based on available data from the Georgia Department of Public Health and the CDC [19, 20, 22–28]. HIV transmission probabilities were obtained from published studies among the MSM population [44–46]. Behavioral data for the MSM population in the community and while incarcerated were obtained from published US studies [47–57]. We also obtained longitudinal clinical data for patients from the British Columbia Centre for Excellence in HIV/AIDS to estimate the plasma viral load trajectory of MSM patients prescribed ART [58, 59].

### Deterministic Compartmental Transmission Model

**Fig 1:**
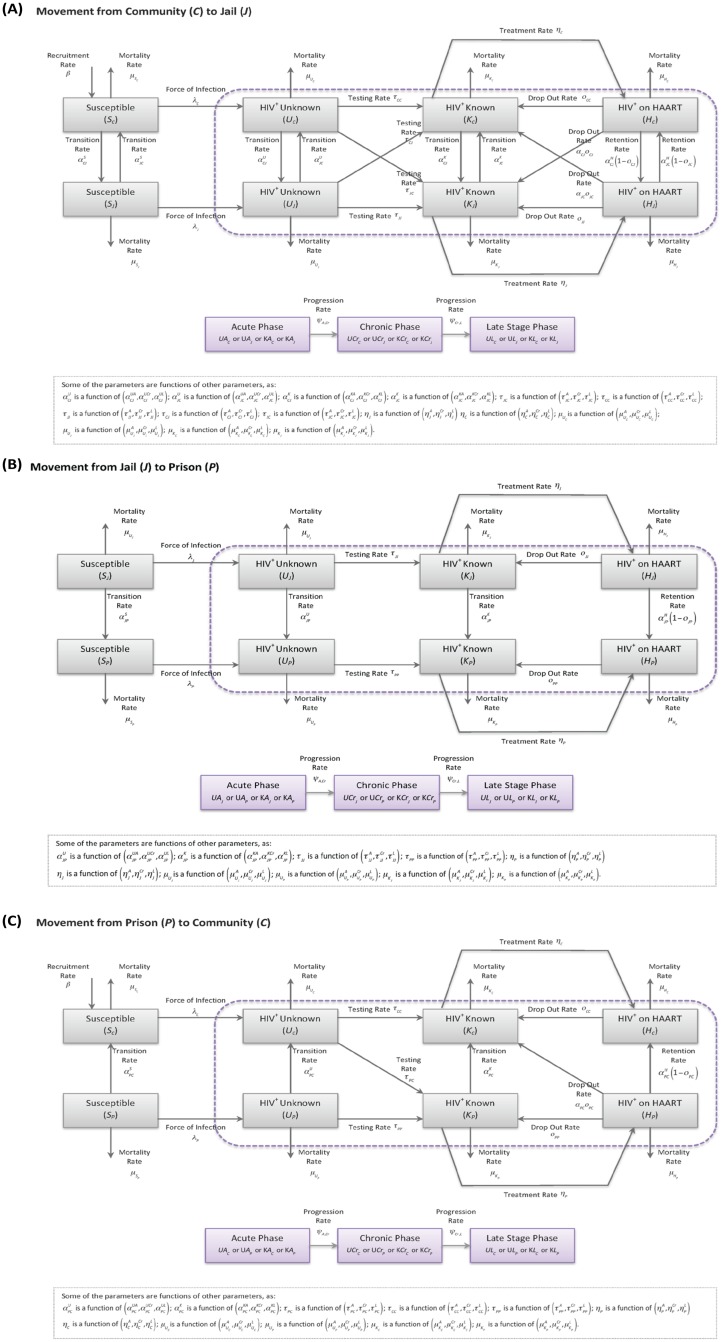
Model Description; (A) Movement between Community (*C*) and Jail (*J*), (B) Movement between Jail (*J*) and Prison (*P*), and (C) Movement between Prison (*P*) and Community (*C*).

The model is presented in Fig 1 and a detailed list and explanation of all parameters can be found in S1 File. Briefly, the model stratified the population according to CJS status into three groups: individuals residing in the community and those incarcerated in jail and in (state) prison. In the US, jails are confinement facilities administered by local law enforcement agencies that detain before or after adjudication. Inmates sentenced to jail facilities usually have a sentence of one year or less. Prisons are long-term confinement facilities run by a state or the federal government that typically hold felons and offenders with sentences of more than one year. Prisons are long-term confinement facilities run by a state or the federal government that typically hold felons and offenders with sentences of more than one year. Note that the community compartment included people on probation or on parole, and we did not explicitly model the effect of recidivism on the risk of acquiring HIV. The possible transitions across settings in the model included Community→Jail, Jail→Community, Jail→Prison and Prison→Community. Thus, in order for an individual go from the community to prison, he needs to go first to jail and then to prison (rarely individuals go directly to prison). Individuals were also stratified according to their stage in the HIV continuum of care as: susceptible (i.e. not infected), HIV-infected but not tested, HIV-infected tested and HIV-infected on ART. In Fulton County in 2012, ART was initiated based on a CD4 cell count threshold of ≤350 cells/mm^3^ and this is the criterion used in the Status Quo scenario. We note that the HIV treatment guidelines have since changed and it is expected that all people be offered ART regardless of CD4. Thus, in all HIV-TTR expansion scenarios we used this new criterion to determine the benefit for the population from making universal treatment available. For individuals in the HIV-infected not tested and tested compartments, disease stages were assigned as follows: acutely infected (up to six months since infection), chronically infected (six months from infection to diagnosis of AIDS), and late stage (if an individual experienced any AIDS-defining condition) [15, 60]. Among those on ART, we divided the population into different plasma viral load strata (<3 log_10_ copies/mL, ≥3 and <4 log_10_ copies/mL, ≥4 log_10_ copies/mL) and in a separate compartment if the individual experienced any AIDS-defining condition after starting ART [58].

**Fig 2:**
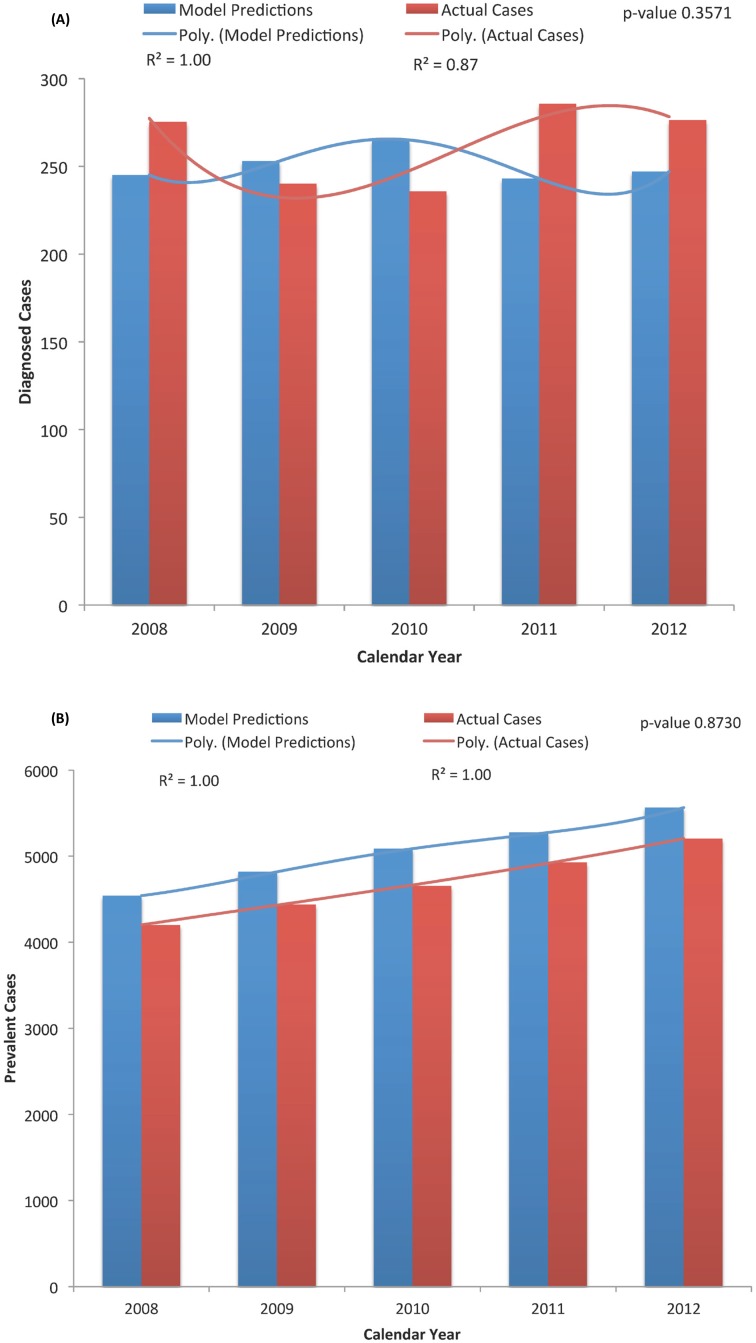
Results for the Model Calibration; (A) New Diagnosed Cases in the Community, and (B) Prevalent Cases in the Community. Actual data represents the new diagnoses (A) or prevalent (B) cases reported by the Georgia Department of Public Health [19, 20, 22–28]. Data were compared using the two-sample Kolmogorov-Smirnov test.

**Fig 3:**
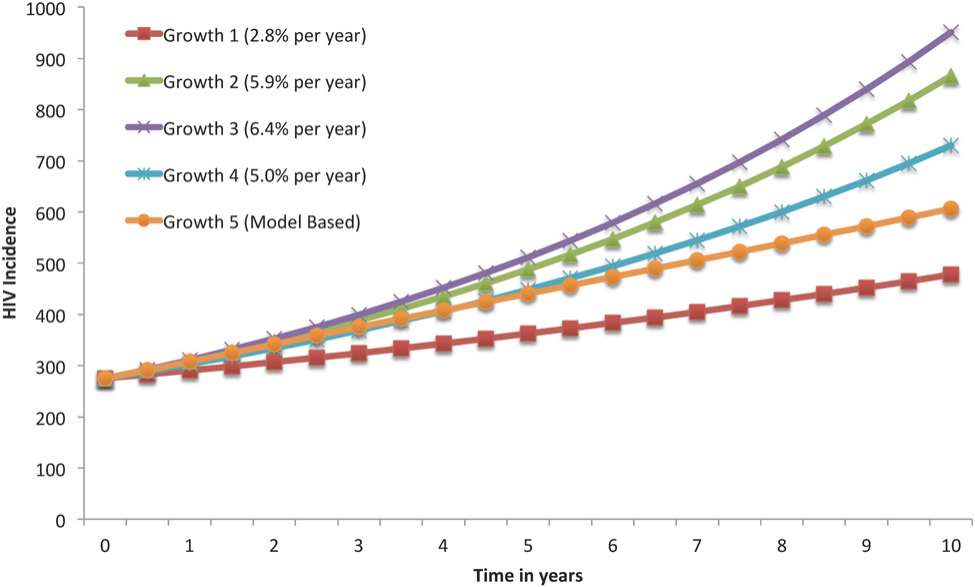
Comparison of HIV Incidence Curves Based on the Present Model and on Other Growth Models Published on the Literature. Growth 1 was based on the results from the HTPN 061 study based on all trial participants [53, 55, 56]; Growth 2 was based on the results from the HTPN 061 study based on trial participants aged 30 years or less [53, 55, 56]; Growth 3 was based on the results from the InvolveMENt study [54]; Growth 4 was based on the average of the previous growth models; Growth 5 was based on this model’s predictions.

This model was calibrated based on available data for HIV prevalence and new positive tests in the community, from 2008 to 2012, obtained from the Georgia Department of Public Health (Fig 2) [19, 22–28]. Note that longitudinal information for these same outcomes for the jail and prison settings were not available at the Bureau of Justice Statistics and, therefore, we relied on expert opinion to assess the validity of our model predictions in these settings. In addition, we compared our model HIV incidence estimates with the results of two studies involving MSM in Atlanta, Georgia—the HTPN 061 and the InvolveMENt studies (Fig 3) [53–56]. In this calibration, we compared the published data with the model predictions using the two-sample Kolmogorov-Smirnov test (Fig 3).

Each model run was the result of a different combination of parameter values, and the model outputs were gathered at every six months for 10 years (2013 to 2022). In this model we assumed a homogeneous mixing between MSM in each of the settings. A more detailed explanation of the differential equations that govern this model, the parameter values for the Status Quo scenario and the model’s initial conditions can be found in S1 File. The model was implemented using Berkeley Madonna version 8.3.13.

### Main Outcomes

We projected the course of the HIV epidemic in Fulton County by comparing the Status Quo scenario to different HIV-TTR scenarios in the community, jail, and prison settings. The measures of intervention impact at the end of 10 years included: (1) the number of averted HIV incident, prevalent and mortality cases; (2) HIV incidence rates; and (3) the percentage reduction in HIV incidence, prevalence and mortality. HIV incident cases are equivalent to the number of newly acquired infections. HIV incidence rates were defined by dividing the estimated HIV incidence by estimates of the size of the susceptible population in a given year. The percent change was defined by calculating the difference in outcome between the intervention and the Status Quo scenario in a given year and dividing this by the same outcome in the Status Quo scenario.

### Modeling Scenarios

In the community, jail and prison, we estimated the effect of: (1) only increasing testing; (2) only increasing ART treatment. Here, we considered the approach of initiating therapy just after receiving a positive test regardless of CD4 count criteria (i.e. a universal test and treat approach); and (3) simultaneously increasing testing, ART treatment coverage and retention in treatment based on a universal test and treat approach. It is important to mention that in both jail and prison, there is a high rate of treatment interruption (i.e. low retention) for individuals returning to the community (only 38% are currently retained in care six months post-release from jail and 30% sixty days after been released from prison) [39, 61]. Therefore, a key intervention in the model was to increase the treatment retention rate upon release from jail or prison.

Note that all rates in each of the scenarios that we describe next are per 100 persons/6 months. The scenarios in each setting were as follows: (1) Community—we increased HIV testing and ART coverage rates to 60, 80 and 100 and retention in treatment rates to 100; (2) Jail—currently, Fulton County has an opt-out rapid HIV testing on jail entry and since most jail episodes are short in length [32, 34, 38], one of the possible interventions was increasing HIV testing rates to 60, 80 and 100. Since some jail detainees have longer jail episodes, however, we also increased ART coverage rate to 60, 80 and 100 and retention in treatment rate to 100. In this model, we did not consider testing once the individual is already in jail since this scenario is rare based on current jail practice; and (3) Prison—we increased ART coverage rate to 60, 80 and 100 and retention in treatment rate from 95 to 100. Note that currently, in Georgia, HIV testing on prison entry is mandatory and effectively at 100% [62]. In this model, we did not consider testing once the individual is already in prison since, in practice, testing does not occur again until prison discharge or by inmate request.

Last, in addition to increasing HIV-TTR rates, we assessed the additional benefit of increasing condom use. We based this analysis on the HIV-TTR coverage rate scenarios 60/60/100, 80/80/100 and 100/100/100 per 100 persons/6months. In each of these scenarios we increased condom use as follows: (1) Community—we increased the condom use from 63% (Status Quo) of all anal sexual acts to 70%, 75%, 80%, 85% and 90%; (2) Jail and Prison—currently in Georgia, there is no sanctioned condom distribution in both jail and prison. However, to assess whether any level of condom distribution in this setting would have any additional effect on HIV incidence and prevalence, we increased the condom use from 0% (Status Quo) of all anal sexual acts to 30%, 60% and 90%.

### Sensitivity Analysis

To evaluate the impact of individual model assumptions, we performed univariate sensitivity analyses using as basis the parameter values from the Status Quo scenario. The impact of the sensitivity analyses was assessed by calculating the percent change in HIV incidence and prevalence in the community, jail and prison at the end of 10 years. Due to the higher uncertainty in our behavior parameters, we decided to run these sensitivity analyses focused on increasing the following parameters, in all settings, by 20%, 40% and 60%: the rates of transmission through receptive and insertive anal sex, the probability of having unprotected receptive anal sex, and the product between the average number of anal sexual partners and anal sexual encounters. We also assessed the impact of condom efficacy on the same model outcomes since the literature reports on a range for efficacies [57, 63]. In this case, we reduced this parameter by 10%, 15% and 20%. Please refer to S1 File for a detailed description of the value of these parameters in the Status Quo scenario.

## Results

### Status Quo Scenario

Table 1 shows the results of HIV new infections, point prevalence and mortality cases generated by the model under the for the Status Quo scenario at the end of ten years (i.e. year 2022). The model predicted that between 2013 and 2022, there would be an estimated 9,246 new infections acquired in the community, 77 new infections acquired in jail, and 154 new acquired infections in prison. The 10-year cumulative number of mortality cases in the community, jail and prison would be 2,585, 18 and 34 cases, respectively. By 2022, the estimated HIV point prevalence would be 10,815, 69 and 152 cases in the community, jail and prison, respectively. Additionally, the model estimated that the HIV incidence rate in year 2022, in the community, jail and prison would be 3.31, 5.00 and 3.94 per 100 susceptible population/year, respectively.

**Table 1:** Population Benefits over 10 Years of Expanded HIV Testing/Treatment/Retention in the Community, Jail and Prison.

Strategy	New HIV Acquired Infections			All-Cause Mortality			HIV Prevalence		
	N	Averted Cases	% Change from Status Quo	N	Averted Cases	% Change from Status Quo	N	Averted Cases	% Change from Status Quo
***Community Setting***									
Status Quo	9246	−	−	2585	−	−	10815	−	−
Expanded Testing Only									
60 per 100 persons/6months	9025	221	-2.39	2514	71	-2.75	10597	218	-2.01
80 per 100 persons/6months	8993	253	-2.74	2503	82	-3.17	10568	247	-2.29
100 per 100 persons/6months	8975	271	-2.93	2495	90	-3.48	10551	264	-2.44
Expanded Treatment Only									
60 per 100 persons/6months	8864	382	-4.13	2278	307	-11.88	10744	71	-0.65
80 per 100 persons/6months	8790	456	-4.93	2241	344	-13.31	10710	105	-0.97
100 per 100 persons/6months	8735	511	-5.53	2218	367	-14.20	10680	135	-1.25
Expanded Testing, Treatment, Retention									
60/60/100 per 100 persons/6months	8071	1175	-12.71	2140	445	-17.21	10062	753	-6.96
80/80/100 per 100 persons/6months	7939	1307	-14.14	2102	483	-18.68	9967	848	-7.84
100/100/100 per 100 persons/6months	7850	1396	-15.10	2077	508	-19.65	9901	914	-8.45
***Jail Setting***									
Status Quo	77	−	−	18	−	−	69	−	−
Expanded Testing Only									
60 per 100 persons/6months	74	3	-3.90	17	1	-5.56	67	2	-2.89
80 per 100 persons/6months	74	3	-3.90	17	1	-5.56	66	2	-3.25
100 per 100 persons/6months	73	4	-5.19	17	1	-5.56	66	2	-3.48
Expanded Treatment Only									
60 per 100 persons/6months	75	2	-2.60	14	4	-22.22	68	0	-0.42
80 per 100 persons/6months	75	2	-2.60	14	4	-22.22	68	0	-0.71
100 per 100 persons/6months	74	3	-3.90	14	4	-22.22	68	1	-0.97
Expanded Testing, Treatment, Retention									
60/60/100 per 100 persons/6months	64	13	-16.88	12	6	-33.33	64	5	-7.38
80/80/100 per 100 persons/6months	63	14	-18.18	12	6	-33.33	63	6	-8.29
100/100/100 per 100 persons/6months	62	15	-19.48	11	7	-38.89	62	6	-8.94
***Prison Setting***									
Status Quo	154	−	−	34	−	−	152	−	−
Expanded Treatment Only									
60 per 100 persons/6months	152	2	-1.30	31	3	-8.82	152	0	-0.16
80 per 100 persons/6months	151	3	-1.95	31	3	-8.82	151	1	-0.46
100 per 100 persons/6months	150	4	-2.60	30	4	-11.76	151	1	-0.73
Expanded Testing, Treatment, Retention									
100/60/100 per 100 persons/6months	143	11	-7.14	29	5	-14.71	143	9	-5.97
100/80/100 per 100 persons/6months	142	12	-7.79	29	5	-14.71	142	10	-6.67
100/100/100 per 100 persons/6months	141	13	-8.44	28	6	-17.65	141	11	-7.16

A detailed explanation for each of the parameter values for the Status Quo scenario can be found in S1 File.

### HIV-TTR Scenarios

#### Community Setting

Results for the impact for each intervention scenario on model outcomes in the community are presented in Table 1. The estimated cumulative number of acquired new infections would be between 9,025 (lowest coverage) and 8,975 (highest coverage) if only Testing coverage was increased (between 221 and 271 averted cases; % decrease from Status Quo between 2.39% and 2.93%); between 8,864 and 8,735 if only Treatment coverage was increased (between 382 and 511 averted cases; % decrease from Status Quo between 4.13% and 5.53%); and between 8,071 and 7,850 if Testing, Treatment and Retention coverage were simultaneously increased (between 1,175 and 1,396 averted cases; % decrease from Status Quo between 12.71% and 15.10%). Focusing now on the scenarios in which we simultaneously increased Testing, Treatment and Retention coverage, we observed that the cumulative number of mortality cases would be between 2,140 and 2,077 cases (between 445 and 508 averted cases; % decrease from Status Quo between 17.21% and 19.65%). The point prevalence after 10 years was estimated to be between 10,062 and 9,901, which is equivalent to a percentage reduction from the Status Quo scenario between 6.96% and 8.45%. Additionally, the model estimated that the HIV incidence rate in year 2022 would be between 2.90 and 2.82 per 100 susceptible population/year.

#### Jail Setting

Results for the impact for each intervention scenario on the model outcomes in jail are presented in Table 1. The estimated cumulative number of new infections would be between 73 (lowest coverage) and 74 (highest coverage) if only Testing coverage was increased (% decrease from Status Quo between 3.90% and 5.19%); between 75 and 74 if only Treatment coverage was increased (% decrease from Status Quo between 2.60% and 3.90%); and between 64 and 62 if Testing, Treatment and Retention coverage were simultaneously increased (% decrease from Status Quo between 16.88% and 19.48%). Focusing now on the scenarios in which we simultaneously increased Testing, Treatment and Retention coverage, we observed that the cumulative number of mortality cases would be between 12 and 11 cases (% decrease from Status Quo between 33.33% and 38.89%). The point prevalence after 10 years was estimated to be between 64 and 62, which is equivalent a reduction from the Status Quo scenario between 7.38% and 8.94%. The model also estimated that the HIV incidence rate in year 2022 would be between 3.34 and 3.24 per 100 susceptible population/year.

#### Prison Setting

Results for the impact for each intervention scenario on the model outcomes in prison are presented in Table 1. Remember that Georgia has 100% testing upon entry into prison and, therefore we only show the results for treatment and retention interventions. The estimated cumulative number of new infections was between 152 (lowest coverage) and 150 (highest coverage) if only Treatment coverage was increased (% decrease from Status Quo between 1.30% and 2.60%), and between 143 and 141 if Testing, Treatment and Retention coverage were simultaneously increased (% decrease from Status Quo between 7.14% and 8.44%). Focusing now on the scenarios in which we simultaneously increased Treatment and Retention coverage, we observed that the cumulative number of mortality cases would be between 29 and 28 cases (% decrease from Status Quo between 14.71% and 17.65%). The point prevalence after 10 years was estimated to be between 143 and 141, which is equivalent a reduction from the Status Quo scenario between 5.97% and 7.16%. The model also estimated that the HIV incidence rate in year 2022 would be between 3.67 and 3.62 per 100 susceptible population/year.

### Testing/Treatment/Retention plus Condom Use in the Community

#### Community Setting

Focusing on the intervention scenarios in which we simultaneously increased Testing, Treatment and Retention coverage, we assessed the additional impact on the estimated HIV cumulative incidence (over 10 years) and the point prevalence (by 2022) if we increased condom use in the community from 63% (condom use in the Status Quo scenario) to 70%, 75%, 80%, 85% and 90% (Table 2). We assessed this impact using the HIV-TTR coverage rate scenarios 60/60/100, 80/80/100 and 100/100/100 per 100 persons/6months. We estimated that the estimated number of new acquired infections for the 60/60/100 scenario for the lowest and highest condom use increase was, respectively, 6,303 cases (1768 averted cases; % decrease from condom Status Quo, 21.91%) and 2,170 cases (5901 averted cases; % decrease from condom Status Quo, 73.12%). In addition, we estimated that the estimated prevalence for the 60/60/100 scenario for the lowest and highest condom use increase was, respectively, 8,594 cases (1,468 averted cases; % decrease from condom Status Quo 14.59%) and 5,176 cases (4,886 averted cases; % decrease from condom Status Quo, 48.56%). The model also estimated that the HIV incidence rate, for the lowest and highest condom use, in year 2022 would be between 2.08 and 0.55 per 100 susceptible population/year. Similar results were obtained for the 80/80/100 and 100/100/100 scenarios.

**Table 2:** Population Benefits over 10 Years of Expanded HIV Testing/Treatment/Retention and Condom Use in the Community.

New HIV Acquired Infections									
Expanded Testing, Treatment, Retention *plus* Condom Use	N			Averted Cases			% Change		
	60/60/100	80/80/100	100/100/100	60/60/100	80/80/100	100/100/100	60/60/100	80/80/100	100/100/100
C (63%), J(0%), P(0%)	8071	7939	7850	−	−	−	−	−	−
C (70%), J(0%), P(0%)	6303	6214	6155	1768	1725	1695	-21.91	-21.72	-21.59
C (75%), J(0%), P(0%)	5133	5070	5028	2938	2869	2822	-36.40	-36.13	-35.94
C (80%), J(0%), P(0%)	4052	4010	3982	4019	3929	3868	-49.80	-49.49	-49.27
C (85%), J(0%), P(0%)	3063	3038	3021	5008	4901	4829	-62.05	-61.74	-61.52
C (90%), J(0%), P(0%)	2170	2156	2147	5901	5783	5703	-73.12	-72.84	-72.65
HIV Prevalence									
Expanded Testing, Treatment, Retention *plus* Condom Use	N			Averted Cases			% Change		
	60/60/100	80/80/100	100/100/100	60/60/100	80/80/100	100/100/100	60/60/100	80/80/100	100/100/100
C (63%), J(0%), P(0%)	10062	9967	9901	−	−	−	−	−	−
C (70%), J(0%), P(0%)	8594	8534	8493	1468	1433	1408	-14.59	-14.37	-14.22
C (75%), J(0%), P(0%)	7624	7585	7559	2438	2382	2343	-24.23	-23.90	-23.66
C (80%), J(0%), P(0%)	6728	6707	6692	3333	3260	3209	-33.13	-32.71	-32.41
C (85%), J(0%), P(0%)	5912	5903	5898	4150	4063	4003	-41.24	-40.77	-40.43
C (90%), J(0%), P(0%)	5176	5176	5177	4886	4790	4724	-48.56	-48.06	-47.71

60/60/100, 80/80/100 and 100/100/100 represent rates (per 100 persons/6months) in the intervention scenarios for Testing/Treatment/Retention. Settings: C—Community, J—Jail and P—Prison. The interventions increased condom use only in the community from 63% to 90%, and maintained condom use in jail and prison at 0%.

#### Jail and Prison Settings

The secondary benefit of increasing condom use in the community was the observed reduction in the estimated new HIV acquired infections and prevalence in both jail and prison. To demonstrate this benefit, we chose the Testing/Treatment/Retention coverage rate scenario 60/60/100 per 100 persons/6months.

In jail, the estimated number of new acquired infections for the lowest and highest condom use increase in the community was, respectively, 60 cases (4 averted cases; % decrease from condom Status Quo, 5.48%) and 49 cases (15 averted cases; % decrease from condom Status Quo, 23.11%). The estimated the HIV prevalence for the lowest and highest condom use increase in the community was, respectively, 55 cases (9 averted cases; % decrease from condom Status Quo, 14.02%) and 34 cases (30 averted cases; % decrease from condom Status Quo, 46.87%). The model also estimated that the HIV incidence rate, for the lowest and highest condom use, in year 2022 would be between 2.89 and 1.79 per 100 susceptible population/year.

In prison, the estimated number of new acquired infections for the lowest and highest condom use increase in the community was, respectively, 139 cases (4 averted cases; % decrease from condom Status Quo 2.79%) and 127 cases (16 averted cases; % decrease from condom Status Quo, 11.13%). The estimated the HIV prevalence for the lowest and highest condom use increase in the community was, respectively, 130 cases (13 averted cases; % decrease from condom Status Quo 9.19%) and 97 cases (46 averted cases; % decrease from condom Status Quo, 32.02%). The model also estimated that the HIV incidence rate, for the lowest and highest condom use, in year 2022 would be between 3.33 and 2.49 per 100 susceptible population/year.

### Testing/Treatment/Retention plus Condom Use in Jail and Prison

Focusing on the intervention scenarios in which we simultaneously increased Testing, Treatment and Retention coverage, we assessed the additional impact on the estimated HIV cumulative incidence (over 10 years) and the point prevalence (by 2022) if we increased condom use in the jail and prison from 0% (condom use in the Status Quo scenario) to 30%, 60% and 90%. We focused on presenting the results for the HIV-TTR coverage rate scenario 60/60/100 for jail and 100/60/100 per 100 persons/6months for prison. Note that the scenarios with higher testing and treatment coverage produced similar results.

In jail, the estimated number of new acquired infections for the lowest and highest condom use increase was, respectively, 46 cases (18 averted cases; % decrease from condom Status Quo, 28.56%) and 9 cases (55 averted cases; % decrease from condom Status Quo, 85.67%) (Table 3). The estimated the HIV prevalence did not change significantly across the condom use scenarios—e.g., for the highest condom use scenario, the prevalence was 63 cases (1 averted case; % decrease from condom Status Quo, 1.57%). The model also estimated that the HIV incidence rate, for the lowest and highest condom use, in year 2022 would be between 2.37 and 0.47 per 100 susceptible population/year.

**Table 3:** Population Benefits over 10 Years of Expanded HIV Testing/Treatment/Retention and Condom Use in Jail.

New HIV Acquired Infections									
Expanded Testing, Treatment, Retention *plus* Condom Use	N			Averted Cases			% Change		
	60/60/100	80/80/100	100/100/100	60/60/100	80/80/100	100/100/100	60/60/100	80/80/100	100/100/100
C (63%), J(0%), P(0%)	64	63	62	−	−	−	−	−	−
C (63%), J(30%), P(0%)	46	45	44	18	18	18	-28.56	-28.87	-28.70
C (63%), J(60%), P(0%)	27	27	26	37	36	36	-57.27	-57.45	-57.35
C (63%), J(90%), P(0%)	9	9	9	55	54	53	-85.67	-85.73	-85.69
HIV Prevalence									
Expanded Testing, Treatment, Retention *plus* Condom Use	N			Averted Cases			% Change		
	60/60/100	80/80/100	100/100/100	60/60/100	80/80/100	100/100/100	60/60/100	80/80/100	100/100/100
C (63%), J(0%), P(0%)	64	63	62	−	−	−	−	−	−
C (63%), J(30%), P(0%)	63	63	62	0	0	0	-0.53	-0.52	-0.52
C (63%), J(60%), P(0%)	63	62	62	1	1	1	-1.05	-1.04	-1.04
C (63%), J(90%), P(0%)	63	62	62	1	1	1	-1.57	-1.55	-1.55

60/60/100, 80/80/100 and 100/100/100 represent rates (per 100 persons/6months) in the intervention scenarios for Testing/Treatment/Retention. Settings: C—Community, J—Jail and P—Prison. The interventions increased condom use only in jail from 0% to 90%, and maintained condom use in the community at 63% and in prison at 0%.

In prison, the estimated number of new acquired infections for the lowest and highest condom use increase was, respectively, 94 cases (49 averted cases; % decrease from condom Status Quo, 34.52%) and 16 cases (127 averted cases; % decrease from condom Status Quo, 88.90%) (Table 4). The estimated the HIV prevalence did not change significantly across the condom use scenarios—e.g., for the highest condom use scenario, the prevalence was 105 cases (38 averted cases; % decrease from condom Status Quo, 26.86%). The model also estimated that the HIV incidence rate, for the lowest and highest condom use, in year 2022 would be between 2.28 and 0.36 per 100 susceptible population/year.

**Table 4:** Population Benefits over 10 Years of Expanded HIV Testing/Treatment/Retention and Condom Use in Prison.

New HIV Acquired Infections									
Expanded Testing, Treatment, Retention *plus* Condom Use	N			Averted Cases			% Change		
	100/60/100	100/80/100	100/100/100	100/60/100	100/80/100	100/100/100	100/60/100	100/80/100	100/100/100
C (63%), J(0%), P(0%)	143	142	141	−	−	−	−	−	−
C (63%), J(0%), P(30%)	94	93	90	49	49	51	-34.52	-34.56	-35.96
C (63%), J(0%), P(60%)	51	51	50	92	91	91	-63.99	-64.02	-64.88
C (63%), J(0%), P(90%)	16	16	15	127	126	126	-88.90	-88.91	-89.20
HIV Prevalence									
Expanded Testing, Treatment, Retention *plus* Condom Use	N			Averted Cases			% Change		
	100/60/100	100/80/100	100/100/100	100/60/100	100/80/100	100/100/100	100/60/100	100/80/100	100/100/100
C (63%), J(0%), P(0%)	143	142	141	−	−	−	−	−	−
C (63%), J(0%), P(30%)	128	127	126	15	15	15	-10.44	-10.45	-10.46
C (63%), J(0%), P(60%)	115	114	114	28	28	27	-19.38	-19.38	-19.40
C (63%), J(0%), P(90%)	105	104	103	38	38	38	-26.86	-26.86	-26.87

60/60/100, 80/80/100 and 100/100/100 represent rates (per 100 persons/6months) in the intervention scenarios for Testing/Treatment/Retention. Settings: C—Community, J—Jail and P—Prison. The interventions increased condom use only in prison from 0% to 90%, and maintained condom use in the community at 63% and in jail at 0%.

### Sensitivity Analysis

**Fig 4:**
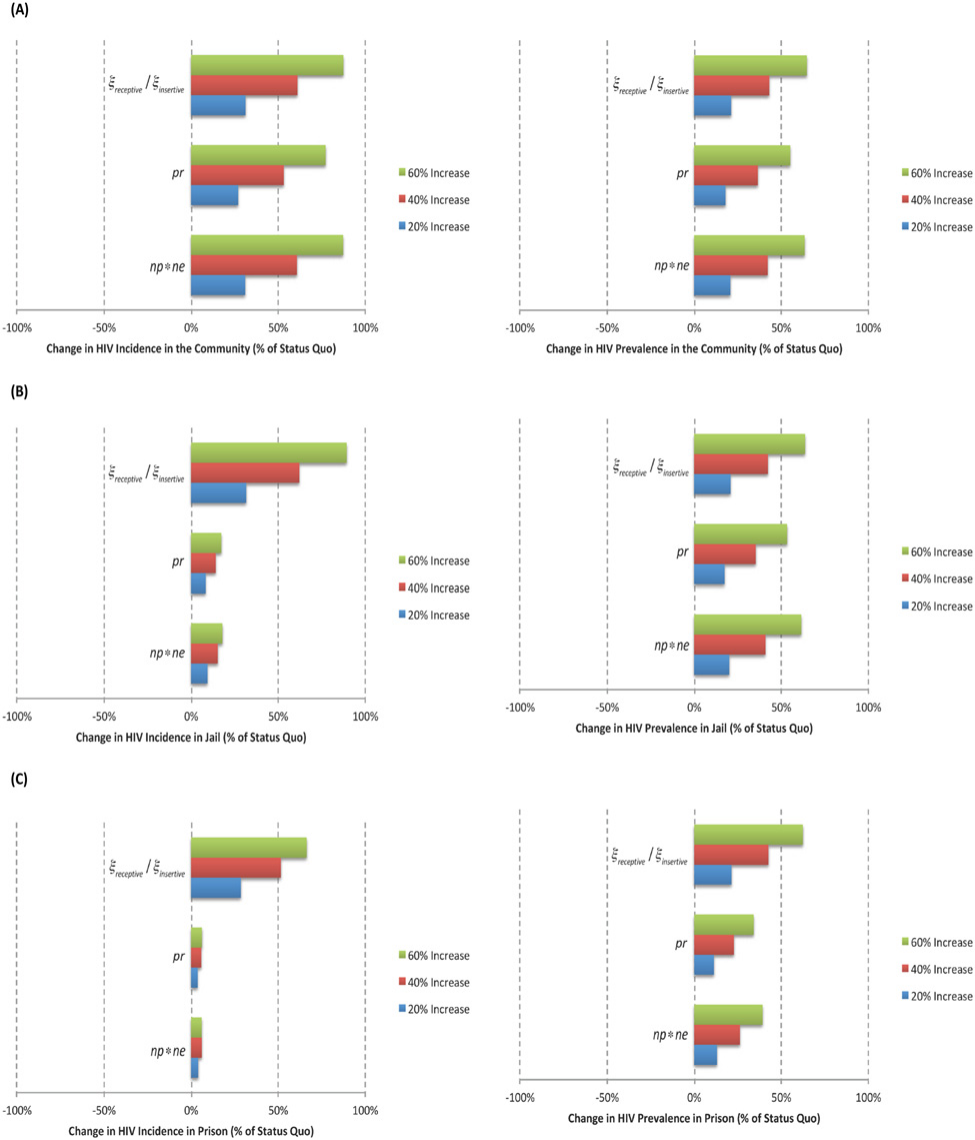
Results from the Univariate Sensitivity Analyses Based on the Percent Change in new HIV acquired infections (i.e. HIV Incidence) and Prevalence from the Status Quo Scenario; (A) Community, (B) Jail, and (C) Prison. This analysis assumed an increase of 20%, 40% and 60% in the rate of transmission through anal receptive (*ξ*_*receptive*_) and insertive (*ξ*_*insertive*_)sex, in the probability of having unprotected receptive anal sex (*pr*), and in the product between the average number of anal sexual partners and anal sexual encounters (np*ne).

**Fig 5:**
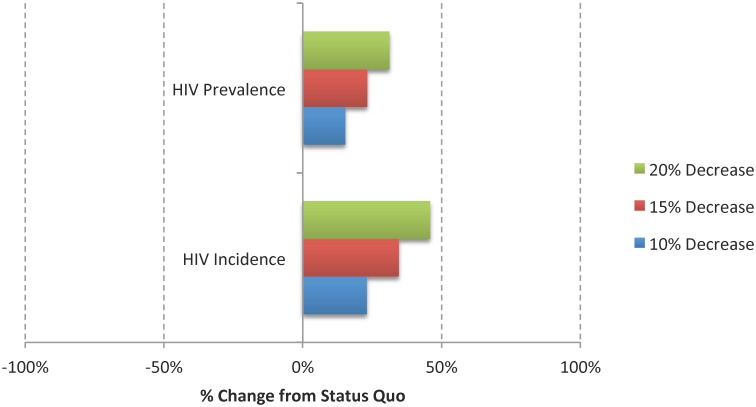
Results from the Univariate Sensitivity Analyses Based on the Percent Change in new HIV acquired infections (i.e. HIV Incidence) and Prevalence from the Status Quo Scenario. This analysis assumed a decrease of 10%, 15% and 20% in condom efficacy (parameter *δ*).

Figs 4 and 5 show the results of the univariate sensitivity analyses on the predicted number of new HIV acquired infections and HIV prevalence for each of the settings.

#### New HIV acquired infections

In the community, these estimates were highly sensitive to the rate of transmission through receptive and insertive anal sex, to the product between the average number of anal sexual partners and anal sexual encounters, and to the probability of having unprotected receptive anal sex, and they were less sensitive to the value used for condom efficacy. In both jail and prison, these estimates were most sensitive to the rate of transmission through receptive and insertive anal sex, and less sensitive to the probability of having unprotected receptive anal sex and to the product between the average number of anal sexual partners and anal sexual encounters.

#### HIV prevalence

In the community, these estimates were highly sensitive to the rate of transmission through anal receptive and insertive sex, to the product between the average number of anal sexual partners and anal sexual encounters, and to the probability of having unprotected receptive anal sex, and they were less sensitive to the value used for condom efficacy. In jail, these estimates were highly sensitive to the rate of transmission through anal receptive and insertive sex and to the product between the average number of anal sexual partners and anal sexual encounters, and they were less sensitive to the probability of having unprotected receptive anal sex. In prison, these estimates were highly sensitive to the rate of transmission through anal receptive and insertive sex, and they were less sensitive to the product between the average number of anal sexual partners and anal sexual encounters, and to the probability of having unprotected receptive anal sex.

## Discussion

We modeled the impact of increasing HIV testing, treatment, and retention in treatment on the HIV epidemic in Fulton County in the black MSM community (at large) and among those who became incarcerated. The model showed that for different scenarios of HIV-TTR we could expect substantial reductions in HIV incidence, prevalence and mortality. The model also showed that in order to maximize the impact of increased testing and treatment coverage in decreasing all model outcomes, retention in treatment in all settings, including the period after jail and prison release, was vital for the success of these interventions. Different studies have pointed out that retention in treatment is essential for the success of any expansion of HIV testing and treatment aimed at preventing future HIV infections [64]. It is important to mention that our model retention parameters encompassed both pre-release and post-release retention in treatment, given that a major issue in this population is the discontinuation of healthcare benefits and treatment upon release from correctional facilities [61, 65–69]. Several studies have demonstrated that the period after release from incarceration can be quite difficult and disruptive since individuals may face several issues including social-economic and health challenges, and in addition, this period may be accompanied by increased sexual behavior [39, 70, 71].

In addition, the model demonstrated that there is a strong additive benefit of expanded condom use in the community setting. In this case, we showed that HIV-TTR interventions alone would not decrease incidence rate below 1 per 100 susceptible population/year at the end of ten years. To decrease the HIV incidence rate below 1 in the community, we needed to significantly increase condom use in the community. These findings independently confirm the results of a mathematical model built to address the MSM epidemic in the United Kingdom [72]. In this case, the authors stressed that condom use should be highly promoted in the population to maximize the benefits of expanded access to HIV testing and treatment. Thus, we stress the importance of implementing interventions involving education and condom distribution to reduce risk behavior in the community and, very importantly, during the critical post-release period. Nationwide, most jails and prisons, including those in Georgia, do not distribute condoms to their inmates, and recently California changed its regulations [73]. Based on the model results, we have shown that lack of access to condoms contributes to the HIV incidence in these settings to remain above 1 per 100 susceptible population/year.

An important consideration in building this model was determining which interventions were feasible in jail and in prison, since they may be highly dependent on the length of stay of different inmates. Among the interventions considered in this study, HIV testing, ART provision and retention in care are highly feasible in prison, since prison episodes are long in nature [74]. In jail, the length of stay is usually short and there is rapid turnover of inmates [32–34]. In this setting, the only in-facility intervention considered was HIV testing. Since some jail detainees have longer episodes, however, in addition to increasing testing, we should also consider increasing ART treatment uptake and encouraging retention into HIV treatment. A recent study conducted in Rhode Island Department of Corrections jail confirmed the feasibility of providing ART treatment when jail incarceration events were greater than 60 days [75]. The extent to which the provision of and retention in treatment may be as successful as when implemented in prison (more stable environment) is unknown though recent data from Connecticut suggests that prescribing ART soon after jail admission results in high levels of viral suppression [74]. It is important to mention that provision of routine HIV screening upon jail or prison entry and ART coverage varies significantly geographically [35, 76–81]; however these settings present the best opportunity to identify undiagnosed infections and to link individuals into care, since for this population, this might be the best opportunity to access proper healthcare services.

Going beyond the mathematical modeling domain, the key question is how to translate the potential that HIV-TTR interventions have in halting the HIV epidemic when dealing with hard-to-reach populations, and how to use these interventions to help reduce racial and social inequalities surrounding the HIV epidemic among correctional populations [82, 83]. One issue not addressed in our model was the effect of recidivism on the risk of HIV infection, which is quite prevalent in Fulton County, Georgia and across the US [61, 82]. Unfortunately, many of these individuals remain at a great risk for not accessing any healthcare upon release and the benefits attained by being on ART can be lost in the long-term, thus promoting HIV transmission [65, 66, 68–70]. Therefore, for these interventions to be successful, the lives of those released from the CJS need to be stabilized through access to housing, employment opportunities, substance abuse treatment, and healthcare entitlements [6, 39, 55, 84]; all of these benefits need to remain in place despite incarceration events.

Our results were based on a mathematical model with important implications and limitations. First, this model is quite intricate because it requires a large number of parameter estimates, and while most parameters were based on empirical data, our behavior parameters were susceptible to a high degree of uncertainty. In particular, caution should be applied when extrapolating these results to other jurisdictions. Another important assumption in this model, mostly for simplification purposes, referred to the transitions between community and CJS. To this end, we assumed that in order for individuals to go to prison they needed to go through jail, and we did not explicitly model transitions among other settings such as probation, parole or alternative programs. We do not believe that modifying the model structure, however, to address this limitation would have a great impact on our projections since these last individuals were modeled as being in the community. Third, we did not model the effect of different comorbid conditions on the risk of HIV acquisition/transmission in this population. It is well known that drug and alcohol addiction, mental illnesses and sexually transmitted infections significantly modulate the risks of incarceration and HIV acquisition/transmission [61, 76, 77]. Although these issues are clearly important determinants of the impact of the HIV epidemic in this population, modeling their effect would greatly increase the model complexity and is beyond the scope of this study. Fourth, in this model we did not consider behavior interventions initiated by the health services staff [85, 86]. To assess the impact of such behavior interventions we can use the results of our analysis, since we showed that the provision and consistent use of condoms are viable interventions to halt the spread of HIV. Fifth, we tried to calibrate our model beyond using prevalence and new cases data in the community. Unfortunately, longitudinal data for several other model outcomes were not available for all settings. Sixth, some studies have reported on as much as 50% reduction in risk behavior upon HIV diagnosis or entry into ART treatment [87, 88]. In this study, we did not consider this additional parameter because the population in these studies was different than ours, although there is some evidence that this reduction in risk could be mirrored in our population [11]. Seventh, the longitudinal viral load distribution was based on the MSM population on ART therapy from a different setting from Atlanta. We chose to take this approach since representative longitudinal data focused on our study population were not available. Eighth, in this model we assumed a homogeneous mixing between MSM in each of the settings. In reality, movement is not random within the confines of a jail or prison, especially for those in solitary confinement. Furthermore, there may not be random mixing in sexual positioning; MSM could have preferences for exclusively insertive or receptive, or versatile behavior. Although mixing is an important component in this type of modeling since it can influence the overall HIV incidence and prevalence, obtaining information on this issue is extremely difficult for our study population and caution should be exercised when making any assumptions in this regard. This limitation highlights the importance of collecting information on MSM sexual mixing preferences in each of the settings so that future models in this population can be more comprehensive. Ninth, the model did not include testing during incarceration, beyond testing done at admission, since this occurs rarely in Fulton County jail or in Georgia’s prisons. However, we built a model flexible enough to assess the effect of testing among inmates if this practice is modified in the future. Finally, it is important to highlight that while we obtained a small yet important, effect on HIV incidence, prevalence and mortality by implementing a HIV-TTR strategy in jail and in prison, we stress that implementing such strategy without focusing on any particular risk group in these settings (e.g., individuals with known high risk behaviour) would have the lowest effect on these outcomes. Therefore, we believe that targeting specific risk groups when implementing a HIV-TTR strategy in jail and in prison would potentially yield the highest effect on these outcomes.

In conclusion, the HIV and incarceration epidemics are highly interconnected and correctional facilities across the US play a major role in providing HIV testing and treatment. The jail and prison settings offer a great opportunity to implementing HIV-TTR strategies to reduce the spread of HIV within the incarcerated population and the community at large. However, the success of this strategy is contingent upon ensuring retention in HIV care after release from jail or prison. This analysis serves as a call to action for public health officials, criminal justice health administrators, and HIV care providers to examine the implementation of Test/Treat/Retain interventions, along with the promotion of condom use among negative and HIV-infected MSM individuals in the community setting. Additionally, we hope that the additional population-level benefit illustrated in this study of condom use in jails and prisons can facilitate future discussions regarding the possibility of introducing condom distribution as another possible harm-reduction strategy in the CJS setting.

## Supporting Information

S1 File Differential Equations, Parameters, Initial Conditions and Data Sources (indexed as per the main text). (PDF)
