# Correction: The Impact of Implementing a Test, Treat and Retain HIV Prevention Strategy in Atlanta among Black Men Who Have Sex with Men with a History of Incarceration: A Mathematical Model

**DOI:** 10.1371/journal.pone.0128734

**Published:** 2015-05-15

**Authors:** Viviane D. Lima, Isabell Graf, Curt G. Beckwith, Sandra Springer, Frederick L. Altice, Daniel Coombs, Bryan Kim, Lauren Messina, Julio S. G. Montaner, Anne Spaulding

The seventh author’s first name is spelled incorrectly. The correct name is: Bryan Kim.
