# Supplementary material for: The Impact of Implementing a Test, Treat and Retain HIV Prevention Strategy in Atlanta among Black Men Who Have Sex with Men with a History of Incarceration: A Mathematical Model
Source: PLoS One. 2015 Apr 23;10(4):e0123482. doi: 10.1371/journal.pone.0123482 (PMC4408043; doi:10.1371/journal.pone.0123482)
Supplement: S1 File — (PDF) [file pone.0123482.s001.pdf]

Supporting Information

Title: The Impact of Implementing a Test, Treat and Retain HIV Prevention Strategy in Atlanta among Black Men Who Have Sex with Men with a History of Incarceration: A Mathematical Model

Authors: Viviane D Lima, Isabell Graf, Curt G. Beckwith, Sandra Springer, Frederick L. Altice, Daniel Coombs, Brian Kim, Lauren Messina, Julio S.G. Montaner, Anne Spaulding; for the Seek Test Treat and Retain Corrections (STTaR Corr) Modeling Group

*To whom correspondence should be addressed. E-mail:vlima@cfenet.ubc.ca

Pages	Section
2 – 3	**Differential Equations**
4 – 12	**Parameters, Initial Conditions and Data Sources (indexed as per the main text)**

Differential Equations

This model was designed to address the 2012 state of the HIV epidemic in Fulton County among persons aged 15-54 years, of black race, and whose risk for acquiring HIV is MSM through unprotected anal intercourse. We stratified the population in this model by setting (community, jail and prison), stage in the HIV continuum of care (susceptible, HIV-positive but not tested, HIV-positive tested and HIV-positive on ART), and HIV disease stage (acutely infected, chronically infected, and late stage). The model is presented in Figure 1 of the manuscript and a detailed list and explanation of all parameters can be found in pages 4 to 11 of this document. This mathematical transmission model was designed to predict the society-wide impact of intensifying prevention interventions including expansion of HIV testing and ART treatment, increased retention into HIV treatment and sexual risk behavior changes. The model is specified by the following set of differential equations:

Susceptible Compartments

$$\frac{\partial S_C}{\partial t} = \beta + \alpha_{JC}^{S} S_J + \alpha_{PC}^{S} S_P - \left(\alpha_{CJ}^{S} + \mu_C^{S}\right) S_C - \frac{\lambda_C S_C}{N_C}$$

$$\frac{\partial S_J}{\partial t} = \alpha_{JC}^{S} S_C - \left(\alpha_{JC}^{S} + \alpha_{JP}^{S} + \mu_J^{S}\right) S_J - \frac{\lambda_J S_J}{N_J}$$

$$\frac{\partial S_P}{\partial t} = \alpha_{JP}^{S} S_J - \left(\alpha_{PC}^{S} + \mu_P^{S}\right) S_P - \frac{\lambda_P S_P}{N_P}$$

HIV-positive/Acute Phase/Not Tested Compartments

$$\frac{\partial UA_C}{\partial t} = \frac{\lambda_C S_C}{N_C} + \alpha_{JC}^{UA}\left(1-\tau_{JC}^{UA}\right) UA_J + \alpha_{PC}^{UA}\left(1-\tau_{PC}^{UA}\right) UA_P - \left(\psi_{A,Cr} + \alpha_{CJ}^{UA} + \tau_{CC}^{UA} + \mu_C^{UA}\right) UA_C$$

$$\frac{\partial UA_J}{\partial t} = \frac{\lambda_J S_J}{N_J} + \alpha_{CJ}^{UA}\left(1-\tau_{CJ}^{UA}\right) UA_C - \left(\psi_{A,Cr} + \alpha_{JC}^{UA} + \tau_{JP}^{UA} + \mu_J^{UA}\right) UA_J$$

$$\frac{\partial UA_P}{\partial t} = \frac{\lambda_P S_P}{N_P} + \alpha_{JP}^{UA}\left(1-\tau_{JP}^{UA}\right) UA_J - \left(\psi_{A,Cr} + \alpha_{PC}^{UA} + \tau_{PP}^{UA} + \mu_P^{UA}\right) UA_P$$

HIV-positive/Chronic Phase/Not Tested Compartments

$$\frac{\partial UCr_C}{\partial t} = \psi_{A,Cr} UA_C + \alpha_{JC}^{UCr}\left(1-\tau_{JC}^{UCr}\right) UCr_J + \alpha_{PC}^{UCr}\left(1-\tau_{PC}^{UCr}\right) UCr_P - \left(\psi_{Cr,L} + \alpha_{CJ}^{UCr} + \tau_{CC}^{UCr} + \mu_C^{UCr}\right) UCr_C$$

$$\frac{\partial UCr_J}{\partial t} = \psi_{A,Cr} UA_J + \alpha_{CJ}^{UCr}\left(1-\tau_{CJ}^{UCr}\right) UCr_C - \left(\psi_{Cr,L} + \alpha_{JC}^{UCr} + \alpha_{JP}^{UCr} + \mu_J^{UCr} + \tau_{JJ}^{UCr}\right) UCr_J$$

$$\frac{\partial UCr_P}{\partial t} = \psi_{A,Cr} UA_P + \alpha_{JP}^{UCr}\left(1-\tau_{JP}^{UCr}\right) UCr_J - \left(\psi_{Cr,L} + \alpha_{PC}^{UCr} + \tau_{PP}^{UCr} + \mu_P^{UCr}\right) UCr_P$$

HIV-positive/Late Stage Phase/Not Tested Compartments

$$\frac{\partial UL_C}{\partial t} = \psi_{Cr,L} UCr_C + \alpha_{JC}^{UL}\left(1-\tau_{JC}^{UL}\right)UL_J + \alpha_{PC}^{UL}\left(1-\tau_{PC}^{UL}\right)UL_P - \left(\alpha_{CJ}^{UL} + \tau_{CC}^{UL} + \mu_C^{UL}\right)UL_C$$

$$\frac{\partial UL_J}{\partial t} = \psi_{Cr,L} UCr_J + \alpha_{CJ}^{UL}\left(1-\tau_{CJ}^{UL}\right)UL_C - \left(\alpha_{JC}^{UL} + \alpha_{JP}^{UL} + \tau_{JJ}^{UL} + \mu_J^{UL}\right)UL_J\left(t\right)$$

$$\frac{\partial UL_P}{\partial t} = \psi_{Cr,L} UCr_P + \alpha_{JP}^{UL}\left(1-\tau_{JP}^{UL}\right)UL_J - \left(\alpha_{PC}^{UL} + \tau_{PP}^{UL} + \mu_P^{UL}\right)UL_P$$

HIV-positive/Acute Phase/Tested Compartments

$$\frac{\partial KA_C}{\partial t} = \tau_{CC}^{UA} UA_C + \alpha_{PC}^{UA}\tau_{PC}^{UA} UA_P + \alpha_{JC}^{UA}\tau_{JC}^{UA} UA_J + \alpha_{JC}^{KA} KA_J + \alpha_{PC}^{KA} KA_P - \left(\psi_{A,Cr} + \alpha_{CJ}^{KA} + \eta_C^{A} + \mu_C^{KA}\right)KA_C$$

$$\frac{\partial KA_J}{\partial t} = \tau_{JJ}^{UA} UA_J + \alpha_{CJ}^{UA}\tau_{CJ}^{UA} UA_C + \alpha_{CJ}^{KA} KA_C - \left(\psi_{A,Cr} + \alpha_{JC}^{KA} + \alpha_{JP}^{KA} + \eta_J^{A} + \mu_J^{KA}\right)KA_J$$

$$\frac{\partial KA_P}{\partial t} = \tau_{PP}^{UA} UA_P + \alpha_{JP}^{KA} KA_J + \alpha_{JP}^{UA}\tau_{JP}^{UA} UA_J - \left(\psi_{A,Cr} + \alpha_{PC}^{KA} + \eta_P^{A} + \mu_P^{KA}\right)KA_P$$

HIV-positive/Chronic Phase/Tested Compartments

$$\frac{\partial KCr_C}{\partial t} = \tau_{CC}^{UCr} UCr_C + \psi_{A,Cr} KA_C + \alpha_{JC}^{KCr} KCr_J + \alpha_{PC}^{KCr} KCr_P + \alpha_{JC}^{UCr}\tau_{JC}^{UCr} UCr_J + \alpha_{PC}^{UCr}\tau_{PC}^{UCr} UCr_P + o_{CC} H_C + \alpha_{PC}^{H} o_{PC} H_P +$$
$$+\alpha_{JC}^{H} o_{JC} H_J - \left(\psi_{Cr,L} + \alpha_{CJ}^{KCr} + \eta_C^{Cr} + \mu_C^{KCr}\right)KCr_C$$

$$\frac{\partial KCr_J}{\partial t} = \tau_{CJ}^{UCr}\alpha_{CJ}^{UCr} UCr_C + \psi_{A,Cr} KA_J + \tau_{JJ}^{UCr} UCr_J + \alpha_{CJ}^{KCr} KCr_C + \alpha_{CJ}^{H} o_{CJ} H_C + o_{JJ} H_J - \left(\psi_{Cr,L} + \alpha_{JC}^{KCr} + \alpha_{JP}^{KCr} + \eta_J^{Cr} + \mu_J^{KCr}\right)KCr_J$$

$$\frac{\partial KCr_P}{\partial t} = \tau_{PP}^{UCr} UCr_P + \psi_{A,Cr} KA_P + \alpha_{JP}^{KCr} KCr_J + \alpha_{JP}^{UCr}\tau_{JP}^{UCr} UCr_J + \alpha_{JP}^{H} o_{JP} H_J + o_{PP} H_P - \left(\psi_{Cr,L} + \alpha_{PC}^{KCr} + \eta_P^{Cr} + \mu_P^{KCr}\right)KCr_P$$

HIV-positive/Late Stage Phase/Tested Compartments

$$\frac{\partial KL_C}{\partial t} = \tau_{CC}^{UL} UL_C + \psi_{Cr,L} KCr_C + \alpha_{JC}^{KL} KL_J + \alpha_{PC}^{KL} KL_P + \alpha_{JC}^{UL}\tau_{JC}^{UL} UL_J + \alpha_{PC}^{UL}\tau_{PC}^{UL} UL_P - \left(\alpha_{CJ}^{KL} + \eta_C^{L} + \mu_C^{L}\right)KL_C$$

$$\frac{\partial KL_J}{\partial t} = \tau_{CJ}^{UL}\alpha_{CJ}^{UL} UL_C + \tau_{JJ}^{UL} UL_J + \alpha_{CJ}^{KL} KL_C + \psi_{Cr,L} KL_C - \left(\alpha_{JC}^{KL} + \alpha_{JP}^{KL} + \eta_J^{L} + \mu_J^{KL}\right)KL_J$$

$$\frac{\partial KL_P}{\partial t} = \tau_{PP}^{UL} UL_P + \psi_{Cr,L} KCr_P + \alpha_{JP}^{KL} KL_J + \alpha_{JP}^{UL}\tau_{JP}^{UL} UL_J - \left(\alpha_{PC}^{KL} + \eta_P^{L} + \mu_P^{KL}\right)KL_P$$

HIV-positive/on HAART

$$\frac{\partial H_C}{\partial t} = \eta_C^{A} KA_C + \eta_C^{Cr} KCr_C + \eta_C^{L} KL_C + \alpha_{JC}^{H}\left(1-o_{JC}\right)H_J + \alpha_{PC}^{H}\left(1-o_{PC}\right)H_P - \left(\alpha_{CJ}^{H} + o_{CC} + \mu_C^{H}\right)H_C$$

$$\frac{\partial H_J}{\partial t} = \eta_J^{A} KA_J + \eta_J^{Cr} KCr_J + \eta_J^{L} KL_J + \alpha_{CJ}^{H}\left(1-o_{CJ}\right)H_C - \left(\alpha_{JC}^{H} + \alpha_{JP}^{H} + o_{JJ} + \mu_J^{H}\right)H_J$$

$$\frac{\partial H_P}{\partial t} = \eta_P^{A} KA_P + \eta_P^{Cr} KCr_P + \eta_P^{L} KL_P + \alpha_{JP}^{H}\left(1-o_{JP}\right)H_J - \left(\alpha_{PC}^{H} + o_{PP} + \mu_P^{H}\right)H_P$$

Table 1. Model Parameters and Initial Conditions.

Parameter	Value	Description	Reference
**Initial conditions**			
$S_C(0)$	16348	HIV-negative (i.e. susceptible) in the community.	[20, 21, 49-56, 76]
$S_J(0)$	114	HIV-negative in jail.	[4, 30, 31, 40-43]
$S_P(0)$	235	HIV-negative in prison.	[4, 30, 31, 40-43]
$UA_C(0)$	1	HIV-positive and not tested (i.e. unknown HIV status) during acute phase in the community.	[1-3, 6, 19, 22-28]
$UA_J(0)$	0	HIV-positive and not tested during acute phase in jail.	[5, 6, 29-43]
$UA_P(0)$	0	HIV-positive and not tested during acute phase in prison.	[5, 6, 29-43]
$UCr_C(0)$	685	HIV-positive and not tested during chronic phase in the community.	[1-3, 6, 19, 22-28]
$UCr_J(0)$	2	HIV-positive and not tested during chronic phase in jail.	[5, 6, 29-43]
$UCr_P(0)$	0	HIV-positive and not tested during chronic phase in prison.	[5, 6, 29-43]
$UL_C(0)$	465	HIV-positive and not tested during late stage phase in the community.	[1-3, 6, 19, 22-28]
$UL_J(0)$	1	HIV-positive and not tested during late stage phase in jail.	[5, 6, 29-43]
$UL_P(0)$	0	HIV-positive and not tested during late stage phase in prison.	[5, 6, 29-43]
$KA_C(0)$	1	HIV-positive and tested (i.e. known HIV status and not on HAART) during acute stage phase in the community.	[1-3, 6, 19, 22-28]
$KA_J(0)$	0	HIV-positive and tested during acute stage phase in jail.	[5, 6, 29-43]
$KA_P(0)$	0	HIV-positive and tested during acute stage phase in prison.	[5, 6, 29-43]
$KCr_C(0)$	913	HIV-positive and tested during chronic stage phase in the community.	[1-3, 6, 19, 22-28]

$KCr_J(0)$	5	HIV-positive and tested during chronic stage phase in jail.	[5, 6, 29-43]
$KCr_P(0)$	10	HIV-positive and tested during chronic stage phase in prison.	[5, 6, 29-43]
$KL_C(0)$	621	HIV-positive and tested during late stage phase in the community.	[1-3, 6, 19, 22-28]
$KL_J(0)$	3	HIV-positive and tested during late stage phase in jail.	[5, 6, 29-43]
$KL_P(0)$	10	HIV-positive and tested during late stage phase in prison.	[5, 6, 29-43]
$H_C(0)$	1150	HIV-positive and on HAART in the community.	[1-3, 6, 19, 22-28]
$H_J(0)$	7	HIV-positive and on HAART in jail.	[5, 6, 29-43]
$H_P(0)$	16	HIV-positive and on HAART in prison	[5, 6, 29-43]
**Recruitment**			
β	Calculated	Recruitment rate into the susceptible African American MSM population, between the ages of 15 and 54 years, in Fulton County, Georgia. This parameter was estimated in the model to maintain a constant population. It was estimated using: $\sum_f \left(S_j\mu_j\right)+\left(UA_f\mu_f^A+UCr_f\mu_f^{Cr}+UL_f\mu_f^L\right)+\left(KA_f\mu_f^A+KCr_f\mu_f^{Cr}+KL_f\mu_f^L\right)+\left(H_f\mu_f^H\right)$, where *f*: Community (*C*), Jail (*J*) and Prison (*P*).	Due to limited data available, we adopted a conservative method.
**Transition between settings**			
α_{CJ}^S	0.0378 plus 10%	Rate of detention from the community to jail among susceptibles. This rate changed due to the calibration.	[5, 6, 29-43]
α_{CJ}^I	0.0378	Rate of detention from the community to jail among all HIV-positive compartments.	[5, 6, 29-43]
α_{JC}^I	5.3692	Rate of release from jail to the community among all HIV-positive compartments.	[5, 6, 29-43]
α_{JP}^S	0.3610 plus 30%	Rate of transfer from jail to prison among susceptibles. This rate changed due to the calibration.	[5, 6, 29-43]
α_{JP}^I	0.3610	Rate of transfer from jail to prison among all HIV-positive compartments.	[5, 6, 29-43]
α_{PC}^I	0.1795	Rate of release from prison to community among all HIV-positive compartments.	[5, 6, 29-43]

**Transitions due to natural history**			
$\psi_{A,Cr}$	3.4286	Rate of disease progression from acute to chronic phase.	[16, 60]
$\psi_{Cr,L}$	0.0714	Rate of disease progression from chronic phase to late stage phase.	[16, 60]
**Transitions related to testing**			
τ_{CC}^{A}	0.0542	Rate of testing in the community for acute phase.	[1-3, 19, 22-28, 52, 78]
τ_{CC}^{Cr}	0.1464	Rate of testing in the community for chronic phase.	[1-3, 19, 22-28, 52, 78]
τ_{CC}^{LS}	0.8663	Rate of testing in the community for late stage phase.	[1-3, 19, 22-28, 52, 78]
$\tau_{CJ}^{A}, \tau_{CJ}^{Cr}, \tau_{CJ}^{LS}$	0.5082	Rate of testing going from the community to jail.	[16, 18, 31-36]
$\tau_{JJ}^{A}, \tau_{JJ}^{Cr}, \tau_{JJ}^{LS}$	0.0000	Rate of testing in jail	Expert (Anne Spaulding)
$\tau_{JC}^{A}, \tau_{JC}^{Cr}, \tau_{JC}^{LS}$	0.0000	Rate of testing going from jail to the community	Expert (Anne Spaulding)
$\tau_{JP}^{A}, \tau_{JP}^{Cr}, \tau_{JP}^{LS}$	1.0000	Rate of testing going from jail to prison	[5, 62], Expert (Anne Spaulding)
$\tau_{PP}^{A}, \tau_{PP}^{Cr}, \tau_{PP}^{LS}$	0.0000	Rate of testing in prison	[5, 62], Expert (Anne Spaulding)
$\tau_{PC}^{A}, \tau_{PC}^{Cr}, \tau_{PC}^{LS}$	0.0000	Rate of testing going from prison to community	[5, 62], Expert (Anne Spaulding)
**Transitions related to treatment initiation**			
$\eta_{C}^{A}, \eta_{J}^{A}, \eta_{P}^{A}$	0.0000	Rate of individuals starting treatment in the community, jail or prison during acute phase.	Expert (Anne Spaulding)
η_{C}^{Cr}	0.5000	Rate of individuals starting treatment in the community during chronic phase.	[19, 22-28] Expert (Anne Spaulding)
η_{J}^{Cr}	0.1700	Rate of individuals starting treatment in jail during chronic phase.	[5, 31-35, 80] Expert (Anne Spaulding)
η_{P}^{Cr}	0.5000	Rate of individuals starting treatment in prison during chronic phase.	[37-39], Expert (Anne Spaulding)
η_{C}^{L}	0.5000	Rate of individuals starting treatment in the community during late stage phase.	[19, 22-28], Expert (Anne Spaulding)
η_{J}^{L}	0.2050	Rate of individuals starting treatment in jail during late stage phase.	[5, 31-35, 80], Expert (Anne Spaulding)

η_P^L	0.5000	Rate of individuals starting treatment in prison during late stage phase.	[37-39], Expert (Anne Spaulding)
**Transitions related to treatment drop-out**			
o_{CC}	0.0500	Rate of individuals dropping out of treatment in the community.	[2, 64], Expert (Anne Spaulding)
o_{CJ}	0.1572	Rate of individuals dropping out of treatment going from community to jail.	[5, 31-37, 75], Expert (Anne Spaulding)
o_{JJ}	0.0250	Rate of individuals dropping out of treatment in jail.	[5, 31-37, 75], Expert (Anne Spaulding)
o_{JC}	0.5438	Rate of individuals dropping out of treatment going from jail to the community.	[31, 37, 38], Expert (Anne Spaulding)
o_{JP}	0.0000	Rate of individuals dropping out of treatment going from jail to prison.	[37, 39, 66], Expert (Anne Spaulding)
o_{PP}	0.0500	Rate of individuals dropping out of treatment in prison.	[37, 39, 66], Expert (Anne Spaulding)
o_{PC}	0.5700	Rate of individuals dropping out of treatment going from prison to the community.	[37, 39, 66], Expert (Anne Spaulding)
**Mortality (HIV-related and non HIV-related)**			
μ_C^S, μ_J^S, μ_P^S	0.0020	Mortality rate of HIV-negative individuals in the community, jail or prison. We assumed the same rate in all settings for lack of more detailed data for jail and prison.	[20, 30], Expert (Anne Spaulding)
μ_C^A, μ_J^A, μ_P^A	0.0020	Mortality rate of HIV-positive individuals in the community, jail or prison during acute phase. We assumed the same rate in all settings for lack of more detailed data for jail and prison.	[5, 19, 20, 22-30, 62], Expert (Anne Spaulding)
μ_C^{Cr}, μ_J^{Cr}, μ_P^{Cr}	0.0128	Mortality rate of HIV-positive individuals in the community, jail or prison during chronic phase. We assumed the same rate in all settings for lack of more detailed data for jail and prison.	[5, 19, 20, 22-30, 62], Expert (Anne Spaulding)
μ_C^L, μ_J^L, μ_P^L	0.0369	Mortality rate of HIV-positive individuals in the community, jail or prison during late stage phase. We assumed the same rate in all settings for lack of more detailed data for jail and prison.	[5, 19, 20, 22-30, 62], Expert (Anne Spaulding)
μ_C^H, μ_J^H, μ_P^H	0.0064	Mortality rate of HIV-positive individuals in the community, jail or prison on HAART. We assumed the same rate in all settings for lack of more detailed data for jail and prison.	[5, 19, 20, 22-30, 62], Expert (Anne Spaulding)

**Force of infection**			
$\lambda_f(t)$	Calculated	It is given by: $\lambda_f(t)=\sum_I \omega_f^I I_f(t)+\sum_{VL}\theta_f^{VL} p_{VL}(t) VL_f(t)$, where *f*: Community (*C*), Jail (*J*) and Prison (*P*); *I*: *UA*, *UCr*, *UL*, *KA*, *KCr*, *KL and H;* and for those on HAART, *VL*: VL^0 : with viral load <3 $\log_{10}$ copies/mL; VL^1 : with viral load ≥3 $\log_{10}$ copies/mL and <4 $\log_{10}$ copies/mL; VL^2 with viral load ≥4 $\log_{10}$ copies/mL; and VL^3 : when the individual has AIDS.	
**Force of infection for those off HAART**			
ω_f^I	Calculated	$\omega_f^I = np_f^I ne_f^I \times (1-\delta co_f^I)(pr_f^I \xi_{receptive}^I + (1-pr_f^I)\xi_{insertive}^I)$; where *f*: Community (*C*), Jail (*J*) and Prison (*P*); *I*: *UA*, *UCr*, *UL*, *KA*, *KCr*, *KL.*	
np_C^A, np_C^{Cr}	12	Average number of anal sexual partners of individuals in the community and infectivity strata *I* during acute and chronic phases. We assumed that individuals have 2 partners per month per 6 months. This number changed due to the calibration since it used to be 13.	[47-56]
np_C^L	Calculated	Average number of anal sexual partners of individuals in the community and infectivity strata *I* during late stage phase. We assumed that in the late stage, this parameter would be half of that in the chronic stage.	[47-56]
np_J^A, np_J^{Cr}, np_P^A, np_P^{Cr}	6	Average number of anal sexual partners of individuals in jail and infectivity strata *I* during acute and chronic phases. We assumed that individuals have 1 partner per month per 6 months.	[48], Expert (Anne Spaulding)
np_J^L, np_P^L	Calculated	Average number of anal sexual partners of individuals in prison and infectivity strata *I* during late stage phase. We assumed that in the late stage, this parameter would be half of that in the chronic stage.	Expert (Anne Spaulding)
ne_C^I	2	Average number of anal sexual encounters of individuals in the community and infectivity strata *I*.	[47-56]
ne_J^I, ne_P^I	2	Average number of anal sexual encounters of individuals in jail and prison and infectivity strata *I*. For lack of better data, we assumed the same number as in the community.	Expert (Anne Spaulding)
$\xi_{receptive}^A$	0.0276	Rate of transmission through anal receptive sex in the acute phase.	[44-46]
$\xi_{receptive}^{Cr}$	0.0114	Rate of transmission through anal receptive sex in the chronic phase.	[44-46]

$\xi^{L}_{receptive}$	0.0228	Rate of transmission through anal receptive sex in the late stage phase.	[44-46]
$\xi^{A}_{insertive}$	0.0019	Rate of transmission through anal insertive sex in the acute phase.	[44-46]
$\xi^{Cr}_{insertive}$	0.0008	Rate of transmission through anal insertive sex in the chronic phase.	[44-46]
$\xi^{L}_{insertive}$	0.0016	Rate of transmission through anal insertive sex in the late stage phase.	[44-46]
pr^{I}_{f}	0.5000	Probability of having unprotected receptive anal sex of individuals in setting *f* and infectivity strata *I*.	[47-56]
co^{I}_{C}	63%	Condom use in the community and infectivity strata *I* – percent of all anal sexual acts.	[47-56]
co^{I}_{J}, co^{I}_{P}	0%	Condom use in jail and prison and infectivity strata *I* – percent of all anal sexual acts.	Expert (Anne Spaulding)
δ	0.9500	Condom efficacy in preventing HIV infection.	[57]
**Force of infection for those on HAART**			
VL^0, VL^1, VL^2, VL^3		Viral load strata (<3 $\log_{10}$ copies/mL, ≥3 and <4 $\log_{10}$ copies/mL, ≥4 $\log_{10}$ copies/mL) plus a separate compartment if the individual was experiencing any AIDS-defining condition after starting ART.	
$np^{VL^0}_{C}, np^{VL^1}_{C}, np^{VL^2}_{C}$	12	Average number of anal sexual partners of individuals in the community and viral load strata *VL=0,1,2.* We assumed that individuals have 2 partners per month per 6 months. This number changed due to the calibration since it used to be 13.	[47-56]
$np^{VL^3}_{C}$	Calculated	Average number of anal sexual partners of individuals in the community and viral load strata *VL=3.* We assumed that in this viral load stratum, this parameter would be half of that in the other viral load strata.	Expert (Anne Spaulding)
$np^{VL^0}_{J}, np^{VL^1}_{J}, np^{VL^2}_{J}$ $np^{VL^0}_{P}, np^{VL^1}_{P}, np^{VL^2}_{P}$	6	Average number of anal sexual partners of individuals in jail and prison and viral load strata *VL=0,1,2.* We assumed that individuals have 1 partner per month per 6 months.	[48], Expert (Anne Spaulding)
$np^{VL^3}_{J}, np^{VL^3}_{P}$	Calculated	Average number of anal sexual partners of individuals in jail and prison and viral load strata *VL=3.* We assumed that in this viral load stratum, this parameter would be half of that in the other viral load strata.	Expert (Anne Spaulding)
ne^{VL}_{C}	2	Average number of anal sexual encounters of individuals in the community and viral load strata *VL*.	[47-56]
ne^{VL}_{J}, ne^{VL}_{P}	2	Average number of anal sexual encounters of individuals in jail and prison and viral load strata *VL*. For lack of better data, we assumed the same number as in	[47-56]

		the community.	
$\xi_{receptive}^{VL^0}, \xi_{receptive}^{VL^1}$	0.0082	Rate of transmission through anal receptive sex in viral load strata *VL=0,1*.	[44-46]
$\xi_{receptive}^{VL^2}$	0.0179	Rate of transmission through anal receptive sex in viral load strata *VL=2*.	[44-46]
$\xi_{receptive}^{VL^3}$	0.0276	Rate of transmission through anal receptive sex in viral load strata *VL=3*.	[44-46]
$\xi_{insertive}^{VL^0}, \xi_{insertive}^{VL^1}$	0.0006	Rate of transmission through anal insertive sex in viral load strata *VL=0,1*.	[44-46]
$\xi_{insertive}^{VL^2}$	0.0013	Rate of transmission through anal insertive sex in viral load strata *VL=2*.	[44-46]
$\xi_{insertive}^{VL^3}$	0.0019	Rate of transmission through anal insertive sex in viral load strata *VL=3*.	[44-46]
pr_f^{VL}	0.5000	Probability of having unprotected receptive anal sex of individuals in setting *f* and viral load strata *VL=0,1,2,3*.	[47-56]
co_C^{VL}	63%	Condom use in the community and viral load strata *VL=0,1,2,3* – percent of all anal sexual acts.	[47-56]
co_J^{VL}, co_P^{VL}	0%	Condom use in jail and prison and viral load strata *VL=0,1,2,3* – percent of all anal sexual acts.	Expert (Anne Spaulding)
p_{VL^0}	0.0160	Probability that individuals at time *t=6 months* will be in viral load strata *VL=0*.	[58, 59] & footnote (3)
p_{VL^1}	0.0398	Probability that individuals at time *t=6 months* will be in viral load strata *VL=1*.	[58, 59] & footnote (3)
p_{VL^2}	0.4272	Probability that individuals at time *t=6 months* will be in viral load strata *VL=2*.	[58, 59] & footnote (3)
p_{VL^3}	0.5170	Probability that individuals at time *t=6 months* will be in viral load strata *VL=3*.	[58, 59] & footnote (3)
p_{VL^0}	0.6166	Probability that individuals at time *t=12 months* will be in viral load strata *VL=0*.	[58, 59] & footnote (3)
p_{VL^1}	0.0412	Probability that individuals at time *t=12 months* will be in viral load strata *VL=1*.	[58, 59] & footnote (3)
p_{VL^2}	0.0992	Probability that individuals at time *t=12 months* will be in viral load strata *VL=2*.	[58, 59] & footnote (3)
p_{VL^3}	0.2430	Probability that individuals at time *t=12 months* will be in viral load strata *VL=3*.	[58, 59] & footnote (3)
p_{VL^0}	0.6599	Probability that individuals at time *t=18 months* will be in viral load strata *VL=0*.	[58, 59] & footnote (3)

p_{VL^1}	0.0456	Probability that individuals at time *t=18 months* will be in viral load strata *VL=1*.	[58, 59] & footnote (3)
p_{VL^2}	0.1134	Probability that individuals at time *t=18 months* will be in viral load strata *VL=2*.	[58, 59] & footnote (3)
p_{VL^3}	0.1811	Probability that individuals at time *t=18 months* will be in viral load strata *VL=3*.	[58, 59] & footnote (3)
p_{VL^0}	0.6613	Probability that individuals at time *t=24 months* will be in viral load strata *VL=0*.	[58, 59] & footnote (3)
p_{VL^1}	0.0517	Probability that individuals at time *t=24 months* will be in viral load strata *VL=1*.	[58, 59] & footnote (3)
p_{VL^2}	0.1223	Probability that individuals at time *t=24 months* will be in viral load strata *VL=2*.	[58, 59] & footnote (3)
p_{VL^3}	0.1648	Probability that individuals at time *t=24 months* will be in viral load strata *VL=3*.	[56, 57] & footnote (3)
p_{VL^0}	0.6392	Probability that individuals at time *t>24 months* will be in viral load strata *VL=0*.	[58, 59] & footnote (3)
p_{VL^1}	0.0443	Probability that individuals at time *t>24 months* will be in viral load strata *VL=1*.	[58, 59] & footnote (3)
p_{VL^2}	0.1586	Probability that individuals at time *t>24 months* will be in viral load strata *VL=2*.	[58, 59] & footnote (3)
p_{VL^3}	0.1579	Probability that individuals at time *t>24 months* will be in viral load strata *VL=3*.	[58, 59] & footnote (3)
p_{VL^0}	0.6155	Probability that individuals at time *t=6 months* will be in viral load strata *VL=0*.	[58, 59] & footnote (3)
p_{VL^1}	0.0440	Probability that individuals at time *t=6 months* will be in viral load strata *VL=1*.	[58, 59] & footnote (3)
p_{VL^2}	0.1819	Probability that individuals at time *t=6 months* will be in viral load strata *VL=2*.	[58, 59] & footnote (3)
p_{VL^3}	0.1586	Probability that individuals at time *t=6 months* will be in viral load strata *VL=3*.	[58, 59] & footnote (3)
**Population**			
$N_f(t)$, $N(t)$	Calculated	$N_f(t)=\sum_I N_f^I(t)$: Sum of all individuals per setting. $N(t)=\sum_f \sum_I N_f^I(t)$: Sum of all individuals in this system.	

Table legend: (1) All rates are reported for 6 months; (2) In this model we considered three settings (denoted by *f* - Community, Jail and Prison), three natural disease phases (denoted by *I* - Acute, Chronic and Late Stage), eight infectivity strata (*S*, *UA*, *UCr*, *UL*, *KA*, *KCr*, *KL and H*), and four

HAART strata (denoted by VL - VL^0 : with viral load <3 $\log_{10}$ copies/mL; VL^1 : with viral load ≥3 $\log_{10}$ copies/mL and <4 $\log_{10}$ copies/mL; VL^2 with viral load ≥4 $\log_{10}$ copies/mL; and VL^3 : when the individual has AIDS; (3) $p_{VL}\left(t\right)$: This is a probability that individuals at time t will be in viral load strata VL. These probabilities were obtained by statistical modeling to estimate the distribution of plasma viral load in the MSM population on HAART at time t. We used Partial Proportional Odds Model (for ordinal responses) to estimate these probabilities. The models were adjusted for demographic and clinical information including: time-varying adherence to HAART, baseline and time-varying CD4 cell count, baseline and time-varying viral load, age, follow-up time, baseline and time-varying AIDS diagnosis, baseline and time-varying class of the HAART regimen. Estimates of HAART adherence were based on dispensed medications, also known as refill compliance estimated, and estimated by dividing the number of months of medications dispensed by the number of months of follow-up.
